# Ripples in the pond: caring for extended family members after a perinatal loss

**DOI:** 10.1186/1471-2393-15-S1-A17

**Published:** 2015-04-15

**Authors:** Nina Bennett, Melanie Chichester

**Affiliations:** 1 Christiana Care Health Services, Newark, Delaware, USA

## 

Parents who have lost a baby during pregnancy, delivery, or shortly after birth are commonly offered time to see their baby, memory items, and support. Grandparents are often forgotten mourners, frequently relegated to supporting the bereaved parents, rather than being recognised as mourners in their own right. Family dynamics can often be severely disrupted following a perinatal loss [1]. Care providers and society in general can expect grandparents to provide comfort to their bereaved adult child, which in effect disenfranchises grandparents from their own grief [2,3]. Grandparents may feel isolated and overwhelmed as they struggle to support their bereaved child while grieving the loss of their grandchild [1]. In challenge to past practice, families are encouraged to remember and talk about their deceased baby [4]. Current theoretical beliefs about grief emphasize the importance of rebuilding meaning as part of the healing process [5].

This paper reported an IRB-approved research study which used a survey to explore how grandparents incorporated the existence of a deceased grandchild into their family history. Eighteen grandmothers completed the survey. Seventeen of the eighteen stated they had pictures of their grandchild. All of them said they conducted some kind of ritual on the birth and/or death day, such as lighting a candle, a balloon release, or a cake at the cemetery. Twelve of the grandmothers wear jewellery that symbolizes their grandchild, and four have had tattoos to help them memorialise their grandchild. Twelve of the eighteen responded that they always include their deceased grandchild when asked how many grandchildren they have, and another five said it depended on the situation.

Although the sample size was relatively small, and homogenous i.e. Caucasian grandparents living in the USA, this survey confirms that grandparents feel a need to memorialize and include their deceased grandchildren into their life story [6]. Implications for practice are that stillbirth has a devastating and disruptive impact on all members of the immediate family including the baby’s grandparents. Therefore bereavement support both at the time of death and later needs to include the extended family.
